# Automated contouring, treatment planning, and quality assurance for total marrow lymphoid irradiation

**DOI:** 10.3389/fonc.2025.1694883

**Published:** 2025-11-17

**Authors:** Eric Simiele, Caressa Hui, Ignacio Romero, Zi Yang, Lawrie Skinner, Lei Xing, Jason B. Ross, Richard T. Hoppe, Michael S. Binkley, Susan M. Hiniker, Nataliya Kovalchuk

**Affiliations:** 1 Radiation Oncology Department, Stanford Healthcare, Stanford, CA, United States; 2 Radiation Oncology Department, The University of Alabama at Birmingham, Birmingham, AL, United States; 3 Radiation Oncology Department, University of California Irvine, Irvine, CA, United States; 4 Radiation Oncology Department, Fresno Cancer Center, Fresno, CA, United States

**Keywords:** TMLI (total marrow lymphoid irradiation), VMAT-TMLI, VMAT-TMI, VMAT-TBI, C-arm linac

## Abstract

**Purpose:**

Total marrow and lymphoid irradiation (TMLI) enables dose escalation to targets while minimizing exposure to surrounding organs at risk (OARs), but its clinical implementation is complex. To simplify this process, contouring, treatment planning, and physics plan checks were automated, and the scripts were made publicly available.

**Methods:**

Fifty patients (age, range 2-64 years) previously treated with volumetric modulated arc therapy total body irradiation (VMAT-TBI) were used for the development of an auto-contouring model to segment the relevant targets. Auto-contours were evaluated using the Dice Similarity Coefficient (DSC), 95% Hausdorff Distance (HD95), and qualitative ranking by four physicians. Automated planning script was created using the Varian Eclipse TPS API and was tested with ten patients: five plans using low-dose 2 Gy TMLI and five plans using high-dose 12 Gy TMLI. Dosimetric parameters, planning time, and blinded physician review were used to evaluate differences between auto and manual plans. Dosimetric differences between the VMAT-TMLI and analogous VMAT- TBI plans were also compared. Plan preparation for treatment and plan check processes were also automated to improve efficiency and to ensure safety and consistency.

**Results:**

The TMLI target auto-contours achieved an average DSC of 0.89 ± 0.03, HD95 of 3.38 ± 1.46, and a reviewers’ ranking of 1.12 ± 0.06, indicating close to “acceptable-as-is”. Compared to the manual VMAT-TMLI plans, the auto-plans demonstrated comparable dosimetric plan quality, with an average dose difference of –1.3% ± 5.9%. Five reviewers (four radiation oncologists and one medical physicist) selected the auto-plans as either equivalent or preferred 74% of the time. However, the required time for the auto-contouring and auto-planning was 4-5 hours compared to an estimated 2-3 days for manual contouring and planning. For both 2 Gy and 12 Gy prescriptions, the VMAT-TMLI plans achieved significantly greater OAR sparing compared to VMAT-TBI, with an average dose reduction of –34.1% ± 9.4%. Notably, the oral cavity, lenses, eyes, and salivary glands exhibited the most significant reductions, each exceeding 50% (all p ≤ 0.05).

**Conclusions:**

An automated VMAT-TMLI planning process was developed, improving efficiency while maintaining clinical quality. The freely available scripts and documentation aim to standardize TMLI delivery and support multi-institutional trials.

## 1 Introduction

Total marrow irradiation (TMI) and total marrow and lymphoid irradiation (TMLI) offers improved OAR sparing for patients undergoing hematopoietic cell transplantation (HCT) by selectively targeting marrow and lymphoid tissue rather than irradiating the entire body as in TBI. These techniques permit dose escalation to potentially improve disease control in relapsed or refractory patients without standard HCT options, reduce OAR exposure in older or comorbid patients, and lower relapse rates in organ or marrow transplant recipients (1–3). The concept of TMI/TMLI was first proposed in 2002 using a Helical Tomotherapy™ system, (TomoTherapy Inc., Madison, WI) (4, 5), with the first patient treated in 2005 (2). City of Hope has an extensive experience with TMI/TMLI using Tomotherapy (now Accuray, Sunnyvale, CA) treating >550 patients since 2005 and has completed 8 prospective trials with acceptable outcomes and toxicity profiles (6, 7). Recently, the City of Hope team showed that TMLI using Volumetric Modulated Arc Therapy on ubiquitous C-arm linacs (VMAT-TMLI) can produce comparable dose distributions as the Tomotherapy technique (8). Although the use of TMLI predates the use of VMAT-TBI, and clinical experience has been well published in several recent clinical (7, 9, 10), and technical reviews (6, 11–13), widespread adaptation of VMAT- TMLI are hampered by concerns of its complexity, which includes extensive contouring and treatment planning (14, 15).

To overcome the complexity associated with VMAT-TMLI, the focus of this work was to automate the contouring, treatment planning, and physics plan check process for VMAT- TMLI. All auto-planning software in this work will be made publicly available to facilitate adoption of the auto-planning workflow by other institutions. To the authors’ knowledge, this is the first comprehensive effort to develop and share an integrated auto-contouring and auto-planning solution for VMAT-TMLI.

## 2 Methods

**Figure 1:**
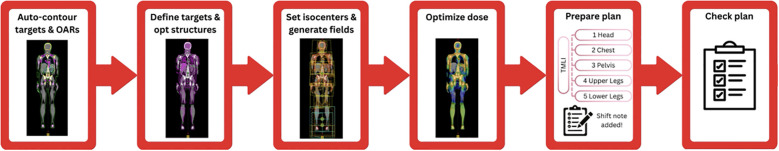
Diagram of the VMAT-TMLI auto-planning workflow. Once the planning CT scan is imported to the treatment planning system, the CT set is exported and the targets and OARs are automatically contoured and imported back to the treatment planning system. The planning target volumes are auto-generated, and subsequently reviewed and approved by physicians. The optimization structures, treatment plans, treatment fields, and optimization objectives are then generated by the auto-planning script. Following review of the generated plans and structures, a second script performs multiple iterations of plan optimizations autonomously. After optimization and physician plan review, the plan is prepared for treatment by separating it into separate plans and generating the shift notes for the therapists. Finally, another script automatically checks various plan elements for completeness and safety.

The workflow for the developed auto-planning process is shown in **Figure 1**. Each step of the process is described in greater detail in the following sections.

### 2.1 Auto-contouring

#### 2.1.1 Training and test data

Auto-contouring was implemented for 35 out of 38 required structures for VMAT-TMLI treatment planning. A dedicated auto-contouring model was developed for three target structures: Bones_Trunk, Bones_Extrem, and LymphNodes. A previously developed auto-contouring model for CSI was used for 22 of the structures (16) including eyes, lenses, parotids, submandibular glands, oral cavity, optic nerves, optic chiasm, larynx, thyroid, esophagus, lungs, heart, kidneys, brain, and spinal canal. The remaining 10 structures were generated using a commercially-available auto-contouring software, Limbus AI (Radformation, New York, NY), and included bowel bag, stomach, bladder, rectum, breasts, uterus cervix, liver, spleen, and mandible. Contouring for three structures not generated by AI models (i.e., ovary right/left and testes) was performed manually. Fifty previously treated VMAT-TBI patients (median, 13.5 years; range, 2–64 years old) were used for the development of the auto-contouring model and divided into 35 training cases, 5 validation cases, and 10 test cases. For each case, the three target structures were manually contoured by a radiation oncologist (LymphNodes) and a medical physicist (Bones_Trunk, Bones_Extrem) to ensure consistency.

#### 2.1.2 Auto-contouring model architecture and training details

A deep-learning model with 3D UNet architecture was selected for the auto-segmentation of three targets using nnUNet 3d fullres configurations (17). Whole body CT scans were used as the model input. The model was trained in 250 epochs with a patch size of 160x160x80 and a batch size of 2 on a single NVIDIA GeForce RTX 4090 GPU using the Dice Similarity Coefficient (DSC) as the loss function. The Adam optimizer (18) was utilized to update the model hyperparameters with the initial learning rates as 1*×*10^-4^.2.1.3 Auto-contouring evaluation metrics.

The DSC was calculated using Equation 1 to evaluate the amount of overlap between the predicted and ground truth contours. The 95th percentile Hausdorff Distance (HD95) was calculated between predicted and ground truth labels to evaluate surface-to-surface distance. A qualitative ranking of auto-contours was also performed by 4 physicians using the clinical trials contour ranking scale: 1 - acceptable, 2 - minor edits, and 3 - major edits.

(1) $$DSC=\frac{2\left|A\cap B\right|}{\left|A\right|+\left|B\right|},$$

### 2.2 Auto-planning

#### 2.2.1 Architecture

Multiple application programming interface (API) scripts were developed within version 15.6 of the Varian treatment planning system Eclipse (Varian Medical Systems, Palo Alto, CA) Scripting API (ESAPI) to facilitate auto-planning. The framework and architecture for these scripts were derived from the results of our previously published works with VMAT- TBI and CSI automated planning (16, 19, 20). The process for auto-planning was broken into two parts, preparation and optimization, to permit the planner to review the created plan, beam placement, and optimization setup (structures and constraints) prior to performing resource-intensive tasks such as optimization and dose calculation. Furthermore, the planner can easily fix any identified issues then rerun the preparation script rather than lose time optimizing plan(s) with a sub-optimal setup.

Once the auto-contours have been reviewed, the planner will launch the preparation script. The user will then select the appropriate plan template for the given patient (templates provided for prescriptions of 2 Gy and 12 Gy). Selecting a plan template will pre-populate all the relevant parameters for preparation. To guide the user in the GUI, the tabs change color depending on the action that should be performed next: red indicates an action should be performed and green indicates the action on that tab is complete. First, the targets are derived based on the treatment regimen for the patient of interest. Target definitions for each TMLI prescription are shown in Equations 2, 3.

(2) $$\begin{array}{l} \text{For }2\text{ Gy prescription}:\\ \text{PTV\_TMLI}=\\ \left[\left(\left(\text{Bones\_Trunk}-\text{Bones\_Face}\right)\;+\text{ LymphNodes }+\text{ SpinalCanal }+\text{ Spleen}\right)\;+\;5\text{ mm}\right]\\ +\left(\text{Ribs }+\;7\;\text{mm}\right)\;+\;\left(\text{Bones\_Extrem }+\;10\;\text{mm}\right)-\left(\text{Lungs }+\;3\;\text{mm}\right)\\ -\left(\text{Kidneys }+\;3\text{ mm}\right)-\left(\text{Eyes }+\;3\text{ mm}\right)-\left(\text{Heart }+\;3\text{ mm}\right)\\ -\left(\text{Esophagus }+\;3\text{ mm}\right)-\left(\text{Larynx }+\;3\;\text{mm}\right)-\left(\text{Ovaries }+\;20\text{ mm}\right) \end{array}$$

(3) $$\begin{array}{l} \text{For }12\text{ Gy prescription}:\\ \text{PTV\_TMLI}=\\ {}[\left(\left(\text{Bones\_Trunk}-\text{Bones\_Face}\right)+\text{LymphNodes}+\text{SpinalCanal}+\text{Spleen}+\text{Testes})+5\;\text{mm}\right]\\ +\left(\text{Ribs }+\;7\;\text{mm}\right)\;+\;\left(\text{Bones\_Extrem }+\;10\text{ mm}\right)-\left(\text{Lungs }+\;3\text{ mm}\right)\\ -\left(\text{Kidneys }+\;3\text{ mm}\right)-\left(\text{Eyes }+\;3\;\text{mm}\right)-\left(\text{Heart }+\;3\text{ mm}\right)\\ -\left(\text{Esophagus }+\;3\text{ mm}\right)-\left(\text{Larynx }+\;3\text{ mm}\right)-\left(\text{Ovaries }+\;20\text{ mm}\right) \end{array}$$

Following target derivation, the structures are sent to the physician for review and approval. Once approved, the planner re-launches the preparation script to generate optimization structures, create the plan, place the beams, and assign optimization constraints. Knowledge-based planning (KBP) is used to generate the auto-plans where plan templates are used as a starting point for plan generation and are modified based on the specific patient anatomy, prescription, etc. All auto-plans contain VMAT isocenters with 2–4 full arcs whereas generated AP/PA plans each contain one isocenter with two opposing static fields. Collimator rotations for the VMAT fields in each isocenter are selected based on templates (3 degrees, 357 degrees, or 90 degrees) except for the pelvis fields (i.e., inferior-most isocenter) where collimator rotations of 0 degrees or 90 degrees are used. Utilizing collimator angles of 0 degrees or 90 degrees simplifies the dosimetric matching between the VMAT and AP/PA portions of the patient’s treatment. All AP/PA fields utilize collimator rotations of 90 degrees.

Once the user is satisfied with the prepared plan, they will launch the optimization loop script. The script will read the “state” of the plan from the preparation script log files and populate the relevant parameters in the UI. The user is provided with one final chance to change the plan objectives and optimization constraints prior to optimization. Once the optimization loop is launched, it proceeds until all plan objectives are met or until the maximum number of user-requested iterations is reached. The optimization loop script was designed to eliminate planner oversight reducing the active effort required for planning these cases.

The auto-planning code developed in this work has been made open-source under the MIT License via Github (https://github.com/esimiele/VMAT-TBI-CSI-TMLI). In addition to the flexibility provided by the script GUIs, configuration and template files are provided with the scripts that can be modified without having to recompile the underlying code. This allows users to easily adapt the scripts to their clinical environment and practice.

#### 2.2.2 Auto-planning evaluation

The developed scripts were tested on ten patients previously treated with VMAT-TBI at our institution: five patients treated to 2 Gy in 1 fraction and five patients treated to 12 Gy in 6 fractions. The VMAT-TMLI plans were manually created to develop the technique for planning and establish achievable constraints based on the experience from City of Hope (11).

The quality of auto-plans was compared to their manual plan counterparts on the basis of clinically relevant DVH metrics such as target coverage, target heterogeneity, intermediate isodose spill, monitor units (MU), etc. Paired t-tests were used to evaluate the significance of any observed differences where a p ≤ 0.05 was considered statistically significant. To further evaluate the quality of the auto-plans, four physicians and one physicist were asked to review the 20 plans in a blinded retrospective manner where all identifying information was removed from the plans. For each patient, the reviewers were asked to choose between the two plans or mark them as equivalent. Finally, the auto- and manual-plans were compared on the basis of the required planning time. The time for manual planning was estimated based on time stamps in Aria/Eclipse whereas the time for auto-planning was determined from evaluating the log files produced from the scripts.

### 2.3 Auto-plan checking

#### 2.3.1 Automated plan checker

The APC tool was adapted from a previously developed ESAPI script that focused on reducing treatment planning errors before they reached patient treatment (21). Liu et al. utilized the Six Sigma DMAIC methodology combined with an FMEA analysis of our institution’s planning and treatment practice to guide development of the APC (21). During testing and initial clinical implementation, they observed a significant reduction in planning errors while simultaneously improving the efficiency of physics plan checking. This tool is still in use at our institution today and is routinely updated based on changes in workflow and planning practice. The present work built on the success of the APC and incorporated checks for identified failure modes during VMAT-TMLI planning. The APC tool was modified and refined during the auto-planning development process and was thoroughly tested for false positives and negatives prior to clinical implementation.

## 3 Results

### 3.1 Auto-contouring

#### 3.1.1 Auto-contouring workflow

The auto-contouring workflow was implemented on a clinical workstation configured to retrieve CT datasets from a designated network location. The auto-planning script interfaces with a DICOM Daemon to initiate the transfer of planning CT scans to this location, at which point the auto-contouring process begins. The segmentation output for targets and OARs is converted from a binary mask into an RT structure set, which is then automatically recognized and imported by the treatment planning system for subsequent use in VMAT-TMLI planning. The entire process is completed in under five minutes.

#### 3.1.2 Auto-contouring evaluation

**Table 1** summarizes the auto-contour performance for three VMAT-TMLI targets. The average DSC was 0.89 *±* 0.03 and HD95 was 3.38 *±* 1.46. Expert qualitative assessments resulted in an average reviewer score of 1.12 *±* 0.06, indicating most contours required minimal or no manual adjustment. The Bones_Extrem structure demonstrated the highest accuracy: DSC 0.92 *±* 0.03, HD95 3.11 *±* 0.93, reviewer score 1.03 *±* 0.08. The accuracy of OAR contours was evaluated in our previous work (16).

**Table 1:** Auto-contouring evaluation metrics for three target structures: Dice Similarity Co-efficient (DSC), 95% Hausdorff Distance (HD95), and a qualitative ranking by four physicians (1 - acceptable, 2 - minor edits, 3 - major edits).

		LymphNodes			Bones_Trunk			Bones_Extrem	
Patient	DSC	HD95	ReviewerRanking	DSC	HD95	ReviewerRanking	DSC	HD95	ReviewerRanking
Patient 1	0.82	5.00	1.50	0.90	1.52	1.00	0.85	4.82	1.00
Patient 2	0.82	5.23	1.50	0.91	1.52	1.25	0.90	3.05	1.00
Patient 3	0.87	4.57	1.00	0.91	1.52	1.00	0.92	2.15	1.00
Patient 4	0.86	5.23	1.25	0.84	3.41	1.50	0.89	4.57	1.25
Patient 5	0.85	5.00	1.25	0.90	3.05	1.00	0.94	3.05	1.00
Patient 6	0.87	5.00	1.00	0.87	3.05	1.25	0.93	3.05	1.00
Patient 7	0.88	5.00	1.25	0.91	1.52	1.00	0.92	3.05	1.00
Patient 8	0.88	5.00	1.00	0.93	2.15	1.00	0.95	3.05	1.00
Patient 9	0.87	5.23	1.25	0.93	1.52	1.00	0.95	2.15	1.00
Patient 10	0.90	4.31	1.25	0.93	1.52	1.00	0.95	2.15	1.00
Average	0.86	4.96	1.23	0.90	2.08	1.10	0.92	3.11	1.03
*σ*	0.03	0.30	0.18	0.03	0.78	0.17	0.03	0.93	0.08

### 3.2 Auto-planning

**Table 2** shows the dosimetric comparison between auto-generated and manually created VMAT-TMLI plans for both 2 Gy and 12 Gy prescriptions. Overall, auto-plans demonstrated moderate improvements in plan quality to the manual plans, with an average OAR and target heterogeneity dose difference of –1.3% ± 5.9%. Statistically significant dose reductions in the auto-planned group were observed for D_mean_ of the kidneys-1cm (–6.6%, p = 0.025), larynx (–3.5%, p = 0.05), thyroid (–3.4%, p = 0.03), and oral cavity (–1.8%, p = 0.03). Bowel D_mean_ was greater in the auto-plans compared to the manual plans (+4.2%, p = 0.01). Difference in MU between auto- and manual plans was not significant (+7.4%, p = 0.08). Overall, for the same plan normalization of 90% PTV_TMLI covered by prescription dose, there was no statistically significant difference in plan inhomogeneity (PTV_TMLI D1cc), dose conformity (Body V50%), or MU between auto- and manual TMLI plans.

**Table 2:** Average dosimetric parameters and differences between auto- and manual VMAT- TMLI plans for 5 test patients treated to 2 Gy and 5 test patients treated to 12 Gy.

DVH metric	2 Gy Rx		12 Gy Rx		% Diff (Auto-Manual)		
	Manual	Auto	Manual	Auto	Ave	σ	p value
Ovaries Dmax [Gy]	1.0	0.9	10.0	6.3	-14.0%	15.5%	N/A
Ovaries Dmean [Gy]	0.8	0.6	6.1	4.5	-10.8%	8.3%	N/A
Kidneys-1cm Dmean [Gy]	1	0.9	5.8	5.3	-6.6%	5.6%	0.025*
Breasts Dmean [Gy]	1.3	1.3	9.5	8.3	-3.6%	5.7%	N/A
Larynx Dmean [Gy]	1.4	1.4	6.6	5.8	-3.5%	5.3%	0.05*
Thyroid Dmean [Gy]	1.1	1.1	9	8.4	-3.4%	4.3%	0.03*
Kidneys Dmean [Gy]	1.2	1.1	7	6.8	-2.4%	4.7%	0.2
Testes Dmean [Gy]	0.6	0.5	13.3	13.1	-2.3%	1.8%	N/A
Lungs Dmean [Gy]	1.2	1.2	7.5	6.9	-1.9%	7.0%	0.09
Cavity Oral Dmean [Gy]	0.4	0.5	3.7	2.8	-1.8%	5.9%	0.03*
Lungs-1cm Dmean [Gy]	0.9	0.9	5.9	5.4	-1.7%	7.7%	0.17
PTV_TMLI D 1.0 cc [Gy]	2.5	2.5	15.2	15.1	-1.1%	5.9%	0.25
Rectum Dmean [Gy]	1.1	1.2	7.1	6.8	-0.7%	3.9%	0.13
Esophagus Dmean [Gy]	1.8	1.8	8.8	9.1	-0.6%	6.3%	0.09
Heart Dmean [Gy]	1.1	1	6.3	6.5	-0.6%	3.4%	0.1
Esophagus Dmax [Gy]	2.3	2.2	12.4	12.7	-0.4%	5.7%	0.15
Parotids Dmean [Gy]	1	1	6.2	6.2	-0.3%	2.5%	0.71
Bowel Dmax [Gy]	2.4	2.4	14.4	14.5	0.0%	2.8%	0.5
Liver Dmean [Gy]	1.1	1.2	7.4	7.1	0.2%	4.4%	0.56
Body V50% [%]	85.5	86.1	89.4	89.4	0.3%	8.3%	0.64
Glnd submands Dmean [Gy]	0.9	0.9	5.4	5.6	1.1%	2.6%	0.06
Stomach Dmean [Gy]	1.1	1.2	7.4	7.3	1.4%	4.9%	0.8
Bladder Dmean [Gy]	1.2	1.2	7	7.1	1.9%	4.2%	0.39
Eyes Dmean [Gy]	1.3	1.1	4.9	6.6	2.5%	16.0%	0.06
Brain Dmean [Gy]	1.4	1.5	8.2	8.6	2.9%	4.1%	0.51
Bowel Dmean [Gy]	1.4	1.4	8.5	9.3	4.2%	4.1%	0.01*
Lenses Dmax [Gy]	1	0.9	4.2	5.5	5.3%	7.4%	0.06
Average					-1.3%	5.9%	

* denotes statistical significance with p ≤ 0.05.

**Table 3** shows the average achieved DVH metrics and differences in OAR sparing be- tween the auto-planned VMAT-TMLI and whole-body VMAT-TBI for 2 Gy and 12 Gy prescriptions. The auto-planned VMAT-TMLI plans achieved significantly greater OAR sparing compared to VMAT-TBI, with an average dose reduction of –34.1% *±* 9.4%. Notably, the oral cavity, lenses, eyes, and salivary glands exhibited the most significant reductions, each exceeding 50% (all p ≤ 0.05). In contrast, maximum dose to the bowel and PTV TMLI D1.0cc were slightly higher in VMAT-TMLI plans with an average increase of 4.4%. Difference in MU between VMAT-TMLI and VMAT-TBI plans was statistically significant (+22.5%, p=0.0005). On average, due to the increased target complexity and the need for additional degrees of freedom, VMAT-TMLI plans utilized a greater number of beams (n=15) compared to VMAT-TBI plans n=13) (p = 0.0003), which would result in approximately two minutes longer delivery time.

**Table 3:** Average dosimetric parameters and differences between auto-planned VMAT-TMLI and VMAT-TBI plans for 5 test patients treated to 2 Gy and 5 test patients treated to 12 Gy.

DVH metric	2 Gy Rx		12 Gy Rx		% Diff (TMLI-TBI)		
	TMLI	TBI	TMLI	TBI	Ave	*σ*	p value
Cavity Oral Dmean [Gy]	0.4	2.1	3.7	12.5	75.0%	5.3%	0 .002*
Lenses Dmax [Gy]	0.9	2	4.2	12	-59.8%	9.10%	0.004*
Ovaries Dmean [Gy]	0.8	2.1	6.1	12.2	-58.4%	12.8%	N/A
Glnd submands Dmean [Gy]	0.9	2.1	5.4	12.7	-57.4%	3.3%	0.002*
Parotids Dmean [Gy]	1	2.1	6.2	12.6	-50.8%	3.5%	0.002*
Eyes Dmean [Gy]	1.3	2.1	4.9	12.3	-49.6%	16.1%	0.005*
Rectum Dmean [Gy]	1.1	2.1	7.1	12.5	-45.2%	4.6%	0.002*
Bladder Dmean [Gy]	1.1	2.1	7	12.5	-45.0%	7.4%	0.002*
Liver Dmean [Gy]	1.1	2.1	7.3	12.3	-43.5%	5.2%	0.002*
Stomach Dmean [Gy]	1.1	2.1	7.3	12.3	-43.5%	6.4%	0.002*
Heart Dmean [Gy]	1.1	1.8	6.3	11.7	-43.3%	10.2%	0.003*
Ovaries Dmax [Gy]	1	2.1	10	12.5	-42.6%	21.6%	N/A
Larynx Dmean [Gy]	1.3	2.1	6.6	12.6	-41.6%	7.2%	0.004*
Thyroid Dmean [Gy]	1.1	1.8	9	12.6	-35.4%	9.2%	0.002*
Bowel Dmean [Gy]	1.3	2.1	8.5	12.3	-33.4%	6.6%	0.002*
Breasts Dmean [Gy]	1.3	2	9.5	11.8	-29.3%	9.5%	N/A
Brain Dmean [Gy]	1.4	1.8	8.2	12.2	-27.0%	9.9%	0.008*
Kidneys Dmean [Gy]	1.2	1.6	6.9	8.3	-21.0%	13.6%	0.001*
Esophagus Dmean [Gy]	1.8	2	8.8	12.3	-20.5%	11.7%	0.01*
Esophagus Dmax [Gy]	2.3	2.2	12.4	13.8	-5.3%	8.3%	0.06
Lungs Dmean [Gy]	1.1	1.2	7.5	7.3	-2.8%	15.2%	0.67
Bowel Dmax [Gy]	2.3	2.3	14.4	13.9	3.3%	4.0%	0.02*
Lungs-1cm Dmean [Gy]	0.8	0.9	5.9	5.5	3.3%	20.4%	0.18
PTV_TMLI D 1.0 cc [Gy]	2.5	2.3	15.2	14.7	4.4%	5.0%	0.025*
Average					-34.1%	9.4%	

* denotes statistical significance with p ≤ 0.05.

**Figure 2:**
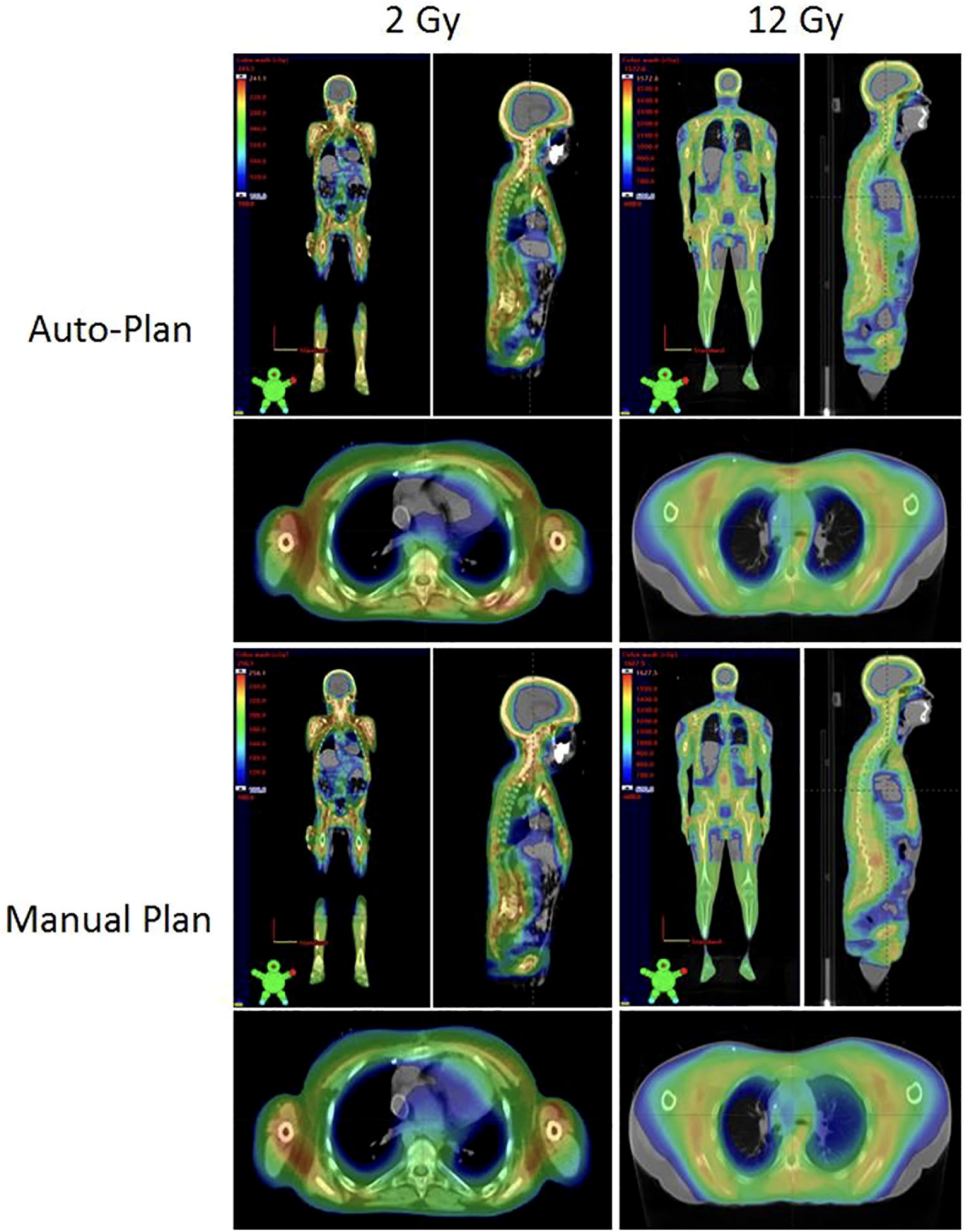
Comparison of the auto-plans and manual plans for adult and pediatric patients for 2 Gy and 12 Gy VMAT-TMLI regimens. The dose clouds have been thresholded to 50% of the prescription dose.

**Figure 3:**
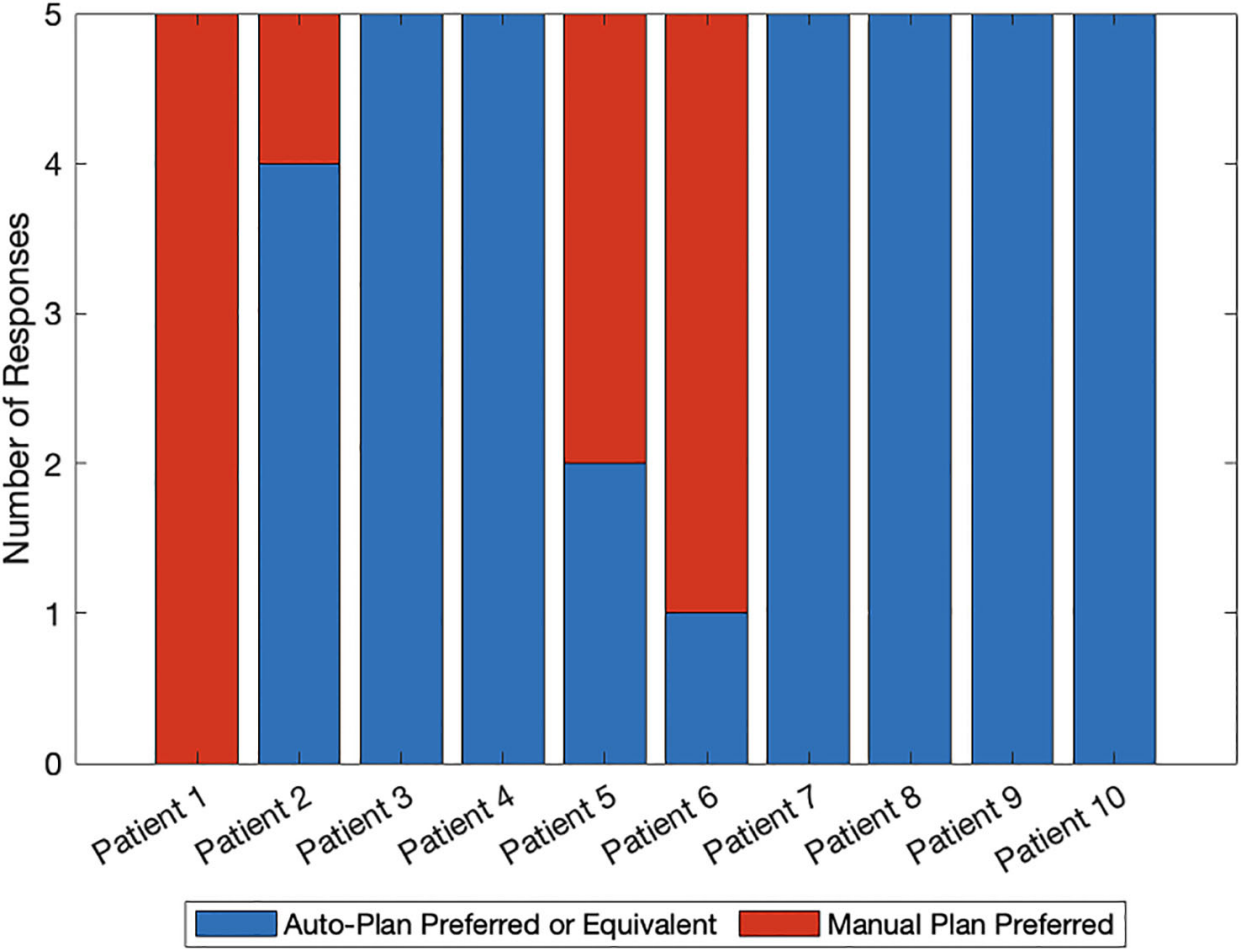
Blinded plan review results by 4 radiation oncologists and 1 medical physicist: blue denotes auto-plan preference or equivalence to the manual plan and red denotes manual plan preference. Patients 1–5 were planned with the 2 Gy prescription regimen whereas patients 6–10 were planned with the 12 Gy prescription regimen.

**Figure 2** shows the comparison between auto- and manual VMAT-TMLI plans for both adult and pediatric patients treated with 2 Gy and 12 Gy prescription doses. The dose cloud has been thresholded to 50% of the prescription dose, and as seen from the axial, coronal, and sagittal slices, the auto plan shows less low-dose spill into the lung and cardiac structures, and less anterior dose spillage into the abdomen compared to the manual plan. Five reviewers (4 radiation oncologists and 1 medical physicist) selected auto-plans as either equivalent or preferred 74% of the time (**Figure 3**). The required time for auto-contouring and planning ranged from 4–5 hours compared to 2–3 days for manual contouring and planning.

### 3.3 Auto-plan checking

#### 3.3.1 Automated plan checker

A sample APC-generated report is available in the **Supplementary Materials**. The report includes patient, course, and plan metadata, followed by itemized checks and corresponding pass/fail statuses. In addition to standard QA parameters (e.g., prescription, dose, energy), the APC evaluates VMAT-TMLI-specific factors such as beam geometry, isocenter positioning, and therapist shift documentation in Aria.

## 4 Discussion

A fully automated pipeline for VMAT-TMLI treatment planning, encompassing auto-contouring, auto-planning, and automated plan checking was developed in this work. The integrated scripting framework was designed to reduce VMAT-TMLI complexity and inter- planner variability and improve workflow efficiency while maintaining high plan quality. The ESAPI auto-planning script is open-source and customizable, enabling other institutions to adapt them to their clinical environment and further refine them for broader use in complex extended-field radiation treatments. Furthermore, the authors hope to encourage multi-institutional studies in cooperative group settings, which was achieved previously for VMAT-TBI where the automated planning VMAT-TBI script developed by our group was incorporated into Children Oncology Group (COG) ASCT2031 multi-institutional clinical trial (22).

The auto-contouring model demonstrated high accuracy across TMLI target structures, with average DSC ranging from 0.86 to 0.92 and average HD95 of 3.38 mm. Reviewer scores consistently indicated minimal need for manual editing. The highest performance was observed in the extremity bones, likely due to their high contrast in CT images and relative anatomic consistency. These results align with previously published work on automated bone and lymph nodes segmentation (23, 24). Watkins et al. (23) developed an auto-segmentation approach using commercial auto-segmentation tool Medical Mind (Medical Mind, Inc., San Diego, CA) for TMI, targeting key planning volumes including: total bone with margin (PTV-Bone), lymph nodes with margin (PTV-Lymph Nodes), the chest wall encompassing ribs and mediastinum (PTV-Ribs), and the skull (PTV-Skull). Reported DSCs ranged from 0.81 to 0.95 across these structures, with HD95 values below 4.5 mm for most targets, except for PTV_Bone, which exhibited a substantially higher HD95 of 30.5 mm. A key distinction of our approach lies in training the AI model to contour clinical target volumes (CTVs) instead of PTVs with margins. This strategy leverages natural tissue-bone boundaries to improve segmentation accuracy and provides greater flexibility for applying institution-specific PTV expansions during planning.

Dei et al. (24) introduced both deep learning (DL) and atlas-based (AB) models to stream- line the TMLI contouring workflow. Their work demonstrated robust OAR segmentation using two DL tools, achieving median DSC of 0.84 and 0.85. For lymph node CTV delineation, the AB approach initially produced a moderate median DSC of 0.70, which improved markedly to 0.94 following manual correction. According to Dei et al., implementing these models reduced the contouring time from 5 to 2 hours. While their results confirm that DL can effectively accelerate OAR segmentation, manual refinement remained necessary for lymph node delineation. In contrast, the present work evaluated the clinical acceptability of target contours through blinded physician review and found that the majority were rated as “acceptable-as-is.” Furthermore, the full auto-contouring process, including both targets and OARs, was completed in under five minutes. Although we did not formally measure the time required for manual contour revisions, these results suggest that the developed pipeline offers both high efficiency and high clinical usability.

Compared to manual VMAT-TMLI planning, automated planning produced dosimetrically equivalent or superior plans with statistically significant reductions in mean doses to several critical organs, including the kidneys, thyroid, larynx, and oral cavity. Importantly, plan comparison by blinded expert reviewers showed a 74% preference or equivalence rating for auto-generated plans. This result reinforces the clinical acceptability of the automated planning method and underscores its potential to streamline treatment planning. Meraldi et al. (25) evaluated the feasibility of using a commercially available knowledge-based planning (KBP) model, RapidPlan (Varian Medical Systems, Palo Alto, CA), to automate VMAT-TMLI planning. Their KB model significantly reduced mean doses to major OARs compared to conventional clinical plans. However, fully automated KB planning (AutoKB) produced elevated target hotspots and suboptimal dose distributions. In contrast, a hybrid approach (HybridKB), which combined KBP with manual refinement, achieved plan quality comparable to expert-generated plans, even when implemented by a planner without prior TMLI experience. A key distinction between the developed auto-planning method and RapidPlan is the optimization strategy: RapidPlan performs only a single round of optimization using the “optimal” optimization constraints (determined by the KB model), while our auto-planning tool executes a minimum of three iterations, with adaptive modification of optimization objectives and constraints to better meet clinical goals. The final iteration is specifically designed to reduce the global hotspot, thereby enhancing target homogeneity and plan quality.

Compared to whole-body VMAT-TBI, the auto-generated VMAT-TMLI plans achieved a 34.1% average reduction in OAR dose, with several structures including the oral cavity, salivary glands, and lenses exhibiting reductions exceeding 50%. These findings are consistent with previous publications on TMLI (7, 9, 11, 12, 26, 27). Importantly, previous studies on VMAT-TBI have highlighted symptomatic mucositis as one of the most common acute toxicities (28–33), with 37% of patients in our institutional experience with VMAT-TBI experiencing grade 3+ mucositis (28). Lower doses to the oral cavity may mitigate this common side effect in patients receiving RT as part of their conditioning regimens for transplantation. Similarly, late side effects including the development of cataracts, infertility, and xerostomia, which are especially pertinent to pediatric patients, may potentially be delayed or spared with the use of TMLI and TMI compared to VMAT-TBI due to decrease doses to OARs.

The automation of optimization and plan checking has the potential to greatly reduce active planning time and improve standardization. While the manual plans required significant planner input and iterative adjustment, the auto-planning scripts achieved similar or better outcomes with minimal manual intervention. Moreover, the integration of an APC specifically tailored for VMAT-TMLI ensures that critical errors, i.e., isocenter misplacement or incorrect shifts, are identified and corrected early in the workflow.

This study has several limitations. First, although the sample size of 10 patients was adequate to demonstrate feasibility and identify statistically significant trends, validation on larger, multi-institutional cohorts is necessary to confirm the generalizability and robustness of the auto-planning framework. Second, while the auto-contouring tool demonstrated high accuracy for target volumes, this study did not re-evaluate the performance of OAR contours, which had been validated previously. Importantly, the auto-contouring tool used in this study is not yet publicly available. Ongoing efforts are focused on collaborating with vendors and partner institutions to synchronize and automate TMLI-specific contouring workflows to enable broader dissemination and integration. Additionally, auto-planning was performed using manually corrected contours rather than auto-generated contours. This approach reflects current clinical practice, where physicians and planners typically review and adjust auto-segmented structures prior to planning. As a future step, we plan to evaluate the dosimetric impact of using uncorrected auto-contours to better understand the implications of a fully autonomous workflow on dose accuracy and clinical acceptability.

Future work will focus on expanding the auto-planning pipeline to support dose- escalated regimens, including 20 Gy protocols for patients with relapsed or refractory leukemia. Single-institution clinical trials are currently in development to evaluate patient outcomes, planning efficiency, and both acute and late toxicities associated with the automated VMAT-TMLI workflow and explore whether the dosimetric differences between VMAT-TMLI and VMAT-TBI treatments translate into clinically meaningful decreases in toxicities. The next phase will involve multi-institutional clinical trials aimed at validating the safety, efficacy, and scalability of TMLI planning across diverse clinical settings.

## 5 Conclusion

The developed auto-contouring, planning, and plan-checking pipeline provide an efficient, high-quality solution for VMAT-TMLI treatment planning. This pipeline has the potential to improve consistency and reduce workload in clinical settings, particularly for complex VMAT-TMLI treatments where planning complexity poses significant challenges in clinical implementation. The auto-planning script has been made publicly available, fostering com- munity collaboration and encouraging broader dissemination. This open-access approach may accelerate the integration of VMAT-TMLI into clinical practice and support the design of multi-institutional trials within cooperative group settings.

## Data Availability

The raw data supporting the conclusions of this article will be made available by the authors, without undue reservation.
