# Supplementary material for: Automated contouring, treatment planning, and quality assurance for total marrow lymphoid irradiation
Source: Front Oncol. 2025 Nov 17;15:1694883. doi: 10.3389/fonc.2025.1694883 (PMC12665524; doi:10.3389/fonc.2025.1694883)
Supplement: Supplementary file 1 [file DataSheet1.pdf]

External Beam Treatment - Physics 2nd Check Report

[redacted] - test - Plan type: VMAT-TMLI

Report Date: 10/27/2025 2:41:51 PM

Prescription Approval	PASS	Rx is approved by MD.
Prescription Dose Per Fraction	PASS	Planned dose per fraction matches linked Rx.
Prescription Fractionation	PASS	Planned number of fraction matches the linked Rx.
Prescription Dose	PASS	Planned dose maches the linked Rx.
Prescription Energy	PASS	Planned energy matches linked Rx for all plans.
Prescription Bolus	PASS	Presence of bolus on all Tx fields if bolus included in Rx.
Prescription Imaging	PASS	Imaging was found in prescription.
Implanted Cardiac Device	PASS	Plan complies with implanted cardiac device policy if applicable.
Current Plan CT	WARN	1 Head: CT image is older than 14 days.
Planning Approval	PASS	planning approved by: Susie Hiniker
CTP note	PASS	CTP note exists for current plans and has been approved by MD.
Patient Orientation	PASS	Tx orientation is correct.
Course Name	PASS	Names are not blank after 'C' character.
Single Active Course	WARN	All courses except for current are completed.
Machine Scale	PASS	Machine IEC61217 scale is used.
Arc Field Name (VMAT)	PASS	ARC field names consistent with direction.
Adequate Tx Time	PASS	Minimum tx time is met.
Dose Rate	PASS	Dose rate set to 200 MU/min for Head and Chest beams and 600 MU/min for Pelvis and Legs.
Tolerance Table	PASS	Tolerance Table set to SHC - VMAT TBI for all plans
Reference Point	WARN	Ref Point C1 TBI 1 Head: Total Dose limit 12.000Gy will be exceeded due to previous dose 12.000Gy resulting in Total Cumulative dose 24.000Gy.
Scheduling Fractions	WARN	Status of 1 or more fractions is not set to 'TREAT'.
Machine Constancy	PASS	All fields and plans have the same Tx machine.
Physics Shifts Check	WARN	Spreadsheet tab for 'AP_PA' was not found!
Matchline Distance Check	PASS	Matchline is equidistant from pelvis and upper leg isos.
Spinning Manny	PASS	Spinning Manny exists in the structure set.
Plan Normalization	PASS	Head, Chest, and Pelvis plans use the same normalization
Jaw Max	PASS	Each jaw does not exceed 20.0cm.
Jaw Min	PASS	Each jaw X & Y >= 3.0cm (3D plan) or 1.0cm (VMAT).
Table Height Check	PASS	Table height is < 17.5 cm. Table height is consistent with other plans.
Table Lateral Check	PASS	Table lateral shift from CT Ref is < 2 cm. Shift is consistent with all plans
Schedule MV imaging	WARN	1 Head: Status of 1 or more images is not set to 'SCHEDULE'.
Schedule kV imaging	WARN	1 Head: Imaging not scheduled for all fractions.
Setup Field Name	PASS	Setup fields named according to gantry angles.
DRR Presence	PASS	High resolution DRRs present for all fields.
MU nonzero	PASS	Treatment fields have nonzero MU.
Setup Field MLC	PASS	Setup fields do not contain MLCs.
Setup Fields Presence	PASS	4 cardinal angle setup fields provided.
Field Isocenter	PASS	All isocenter coords. for fields match.
MLC Check (VMAT/confArc)	PASS	MLC is 'VMAT' or 'Arc Dynamic'.
Collimator check	WARN	Pelvis field has nonzero collimator rotation. Cannot be matched with AP/PA Leg fields

[redacted]
